# Autophagy: A Double-Edged Sword in the Aging of *C. elegans*

**DOI:** 10.3390/ijms27083488

**Published:** 2026-04-14

**Authors:** Tímea Sigmond, János Barna

**Affiliations:** 1 Department of Genetics, Institute of Biology, Eötvös Loránd University, H-1117 Budapest, Hungary; barna.janos@ttk.elte.hu; 2 HUN-REN-ELTE Genetics Research Group, Eötvös Loránd University, H-1117 Budapest, Hungary; 3 Food and Wine Research Institute, Eszterházy Károly Catholic University, H-3300 Eger, Hungary

**Keywords:** autophagy, aging, *Caenorhabditis elegans*, lifespan regulation, proteostasis, stress response

## Abstract

Autophagy is a tightly regulated catabolic process essential for cellular homeostasis, stress adaptation, and regeneration. In the nematode *Caenorhabditis elegans*, with its short lifespan, transparent body, and well-defined genetics, the process can be investigated in a tissue- and age-specific manner, making it an excellent model to study the connection between autophagy and longevity. While autophagy is generally protective—promoting cellular maintenance and longevity—its dysregulation or hyperactivation during aging can be deleterious, leading to cellular stress, tissue damage, and cell death. In this context, autophagy can act as a double-edged sword: its beneficial effects can become harmful if hyperactivated or improperly controlled, particularly in post-reproductive or stressed tissues. Here, we review studies in *C. elegans* that link autophagy to lifespan regulation, with a focus on unexpected, context-dependent, or harmful effects of modulating autophagy-related genes during aging. We highlight how age- and tissue-specific regulation of autophagy can optimize its protective role and discuss the implications of these findings for designing strategies to promote healthy aging, potentially providing insights for the therapeutic targeting of autophagy in humans.

## 1. Introduction

Macroautophagy, or autophagy, is a conserved catabolic process in eukaryotic cells that delivers misfolded proteins, aggregates, and defective organelles to lysosomes via double-membraned autophagosomes for degradation and recycling [1]. Autophagy occurs at a basal level in most tissues under normal physiological conditions, and is rapidly upregulated in response to various intra- and extracellular stressors, such as nutrient deprivation or environmental stress [2]. Dysfunctional autophagy or impaired lysosomal breakdown has been implicated in the pathogenesis of multiple diseases, including inflammation, cardiovascular disorders, and metabolic diseases such as diabetes, cancer, and neurodegeneration. Beyond disease, autophagy is also recognized as a critical regulator of organismal lifespan [3].

**Figure 1:**
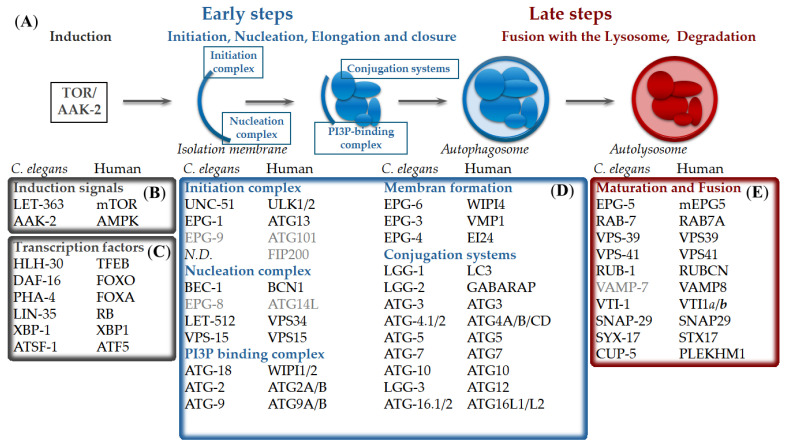
Mechanism and regulation of macroautophagy in *Caenorhabditis elegans* and humans. (**A**) In the early steps of autophagy, cytoplasmic material is sequestered within an isolation membrane, forming autophagosomes. In the late steps, it fuses with lysosomes to degrade content. (**B**) Autophagy induction is regulated primarily by TOR (LET-363) and AMPK (AAK-2). (**C**) Transcription of autophagy-related genes is mainly governed by HLH-30 and other transcription factors. (**D**,**E**) Autophagy-related proteins assemble into hierarchical functional complexes to coordinate initiation, nucleation, vesicle conjugation, maturation, and fusion. N.D. (no data) indicates the absence of a known ortholog. Functional homologs are indicated in gray. The figure is adapted from [5,6].

Macroautophagy proceeds through a series of conserved molecular steps. During induction, parts of the cytoplasm are sequestered by a double-membrane structure known as the isolation membrane, or phagophore. The subsequent closure of the phagophore forms an autophagosome, which then fuses with acidic lysosomes to generate an autolysosome, where the cytoplasmic material is degraded. This process is coordinated by autophagy-related (ATG) proteins that assemble hierarchically into functional complexes (Figure 1A) [4].

Mammalian macroautophagy is initiated by the mammalian target of rapamycin complex 1 (mTORC1) (Figure 1B), which activates the ULK1–ATG13–FIP200–ATG101 initiation complex. The activated ULK1 complex then recruits the Beclin1–VPS34 (class III PI3K) complex (BECN1–PIK3C3–PIK3R4–ATG14) [4,7]. This recruitment leads to PI3P production and omegasome formation at multiple sites of the cytoplasm [8]. PI3P-binding WD-repeat protein Interacting with Phosphoinositides (WIPI) proteins, particularly WIPI2, promote assembly of the ATG12–ATG5–ATG16L1 conjugation complex. This complex facilitates the lipidation of LC3 (MAP1LC3) and GABARAP, which drives phagophore elongation and cargo sequestration. Membrane expansion is thought to involve endosomal reticulum (ER)-derived membranes, ATG9A-positive vesicles, and lipid transfer mediated by ATG2 (Figure 1D) [9]. After closure, the mature autophagosome fuses with lysosomes via RAB7A, the HOPS complex, and STX17–SNAP29–VAMP8 SNARE machinery (Figure 1E) [10]. The material is degraded in the autolysosome by acid hydrolases and recycled [11].

Bulk autophagy is a non-selective process that provides the cell with essential macromolecules, especially under nutrient-deprived conditions [9]. In contrast, selective autophagy, such as mitophagy, aggrephagy, or lipophagy, involves cargo recognition by specific receptors and plays a key role in maintaining cellular quality control and homeostasis [12]. The molecular mechanisms and functions of autophagy are conserved and have been extensively studied in several model organisms, including yeast, flies, mice, and the nematode *Caenorhabditis elegans* [13].

Given its short lifespan, genetic tractability, and conserved autophagy machinery, *C. elegans* has emerged as a powerful in vivo model to dissect the role of autophagy in lifespan regulation [14]. Genetic screens in this nematode have identified several metazoan-specific autophagy genes (e.g., *epg-2*, *epg-3*, *epg-4*, and *epg-5*), expanding our understanding beyond yeast models [14,15]. Studies in this organism have revealed both longevity-promoting functions of autophagy and unexpected age- and tissue-specific detrimental effects, providing a unique framework to understand the context-dependent nature of autophagy during aging [16,17,18,19].

## 2. *C. elegans* as a Model Organism to Study Autophagy

In *C. elegans*, many mammalian autophagy-related proteins have a single ortholog, and genetic studies support the existence of a conserved core machinery [20]. Despite this overall conservation, several notable differences exist between nematodes and mammals. The nematode lacks clear sequence orthologs of certain initiation complex components, such as FIP200, ATG14, and ATG101. Furthermore, although autophagosome–lysosome fusion is SNARE-dependent in both systems, the specific components differ, with mammals primarily utilizing VAMP8, whereas *C. elegans* employs VAMP-7 as the R-SNARE [5]. In addition, key protein families are less diverse in *C. elegans*: the LC3 family and GABARAPs are represented by LGG-1 and LGG-2, while WIPI-related functions are fulfilled by ATG-18 and EPG-6 [5,6]. The core molecular machinery of macroautophagy has been thoroughly reviewed elsewhere [5]. Its mechanism is summarized schematically here, indicating the human orthologs (Figure 1B–E). Beyond bulk degradation, multiple forms of selective autophagy operate in *C. elegans*. Aggrephagy is mediated by the adaptor protein SQST-1/p62, and mitophagy is mediated by the protein DCT-1/BNIP3 [21,22].

Evolutionarily conserved signaling pathways that regulate longevity also control autophagy in worms. The LET-363/TOR (target of rapamycin) pathway coordinates nutrient sensing, protein synthesis, and autophagy (Figure 1B) [23,24]. The insulin/IGF-1 pathway influences metabolism, stress resistance, and lifespan [25,26]. The pathway regulates autophagy in part through the TOR complex and modulates autophagy gene expression via the DAF-16/FOXO transcription factor (Figure 1C) [25].

Longevity-associated processes also induce autophagy. Upon reduction in mitochondrial activity, mitochondrial stress induces AMPK, indicating that the mechanism can be linked to autophagy activation [27]. Dietary restriction (DR) robustly induces autophagy via multiple conserved mechanisms. These include the suppression of the LET-363/TOR and the insulin/IGF-1 signaling, sirtuin activation, epigenetic modulation, and mitochondrial remodeling. Moreover, increased autophagic activity has been observed in eating-defective mutant *C. elegans*, such as *eat-2*, particularly in intestinal tissues [25].

Transcriptional regulation further integrates autophagy with longevity pathways. The helix–loop–helix transcription factor HLH-30, an ortholog of mammalian TFEB, is a central transcriptional regulator of autophagy-related genes in *C. elegans*. Under conditions such as starvation or infection, HLH-30 translocates into the nucleus where it activates the expression of genes involved in autophagy and lysosomal function (Figure 1C) [28,29]. Furthermore, the transcription factor PHA-4/FOXA plays a role in regulating the expression of autophagy-related genes in adult stages, particularly under conditions of dietary restriction or TOR suppression (Figure 1C) [30]. Autophagy gene regulation is also modulated by additional pathways, including TGF-β (Sma/Mab), Rb (LIN-35), the unfolded protein response of the ER (XBP-1), and mitochondrial stress response (ATFS-1) [31], underscoring the multilayered regulation of autophagy during aging (Figure 1C).

### 2.1. Detection and Functional Assessment of Autophagy in C. elegans

Autophagy in *C. elegans* can be visualized in vivo using molecular reporters enabled by the organism’s transparency. To assess different stages of the pathway, several reporters have been developed, including DFCP-1::GFP (omegasome formation), BEC-1::GFP (initiation), GFP::ATG-5 (conjugation machinery), and the GFP::LGG-1 reporter for autophagosome visualization [32,33,34]. Selective cargo turnover can be monitored using GFP::SQST-1, where GFP accumulation indicates defective autophagy [35]. Tandem reporters (GFP::mCherry::LGG-1 or GFP::mCherry::SQST-1) allow for flux assessment by distinguishing autophagosomes from autolysosomes [32,36]. CRISPR/Cas9-based genome editing enables precise endogenous protein tagging, offering clear advantages over traditional multicopy transgenic reporters in *C. elegans*. These endogenous reporters (e.g., GFP::mCherry::LGG-1) minimize artefacts from non-physiological expression, reduce toxicity, and allow more accurate visualization of autophagy dynamics under native regulatory conditions [32]. Reporter assays are commonly complemented by ultrastructural analyses, such as transmission electron microscopy, the gold standard for identifying autophagic compartments [37,38].

Interpretation of autophagy measurements requires caution, as elevation of autophagic structures does not necessarily indicate increased degradation. Importantly, a distinction should be made between autophagy initiation, referring to autophagosome formation, and autophagic flux, which encompasses the entire process from autophagosome biogenesis to lysosomal degradation of cargo. Consequently, increased puncta formation may reflect either enhanced autophagosome formation or autophagosome accumulation due to impaired lysosomal degradation [32,39].

While fluorescent reporters and ultrastructural analyses reveal the formation and identity of specific autophagic compartments, Western blotting provides a complementary readout of autophagic dynamics across the whole organism. LGG-1 lipidation and SQST-1 turnover are commonly used biochemical markers [40,41]. GFP-cleavage assays (e.g., GFP::LGG-1) further enable the assessment of lysosomal delivery, as free GFP accumulates upon cargo degradation in the lysosome [42].

Traditionally, functional studies of autophagy in *C. elegans* have relied on loss-of-function or deletion mutants, providing a robust genetic framework for pathway dissection [43]. More recently, CRISPR/Cas9-based genome editing allows targeted gene knockouts and allele-specific mutagenesis, facilitating the generation of human-disease-relevant models [44,45].

In addition to mutational inactivation, silencing of autophagy genes is also highly accessible in this animal model. RNA interference (RNAi) can be induced by feeding animals bacteria expressing double-stranded RNA that targets specific genes [46]. Sensitivity to RNAi can be enhanced in *rrf-3* mutant backgrounds [47], whereas tissue-specific knockdown is achieved using *sid-1* mutants (defective in systemic RNA interference) complemented with transgenic, cell-type-specific expression of the SID-1 dsRNA receptor [48,49]. The auxin-inducible degradation (AID) system provides a recently developed approach in *C. elegans* for conditional, tissue-specific, and temporally controlled protein depletion, complementing genetic and RNAi-based perturbations [50].

Pharmacological tools provide an additional layer to dissect autophagy dynamics. Lysosomal inhibitors, such as bafilomycin A1 and chloroquine, impair lysosomal acidification and degradation, leading to the accumulation of autophagic intermediates [18,32,51]. In contrast, early-stage inhibitors like 3-methyladenine block autophagy initiation via class III PI3K [52]. Together, these approaches allow for precise, context-dependent analysis of autophagy across tissues and conditions.

Due to its short lifespan (approximately 2–3 weeks under standard laboratory conditions), *C. elegans* provides a unique experimental advantage for autophagy–longevity research. It allows full lifespan analyses within a practical timeframe, allowing direct assessment of how genetic or tissue-specific manipulation of autophagy influences survival across the entire aging trajectory [53,54]. It also displays the conserved aging hallmarks, including muscle deterioration, intestinal and gonadal atrophy, lipid accumulation, and loss of proteostasis and mobility; features that parallel human aging [54]. In conclusion, *C. elegans* offers an unparalleled model for studying the contributions of autophagy in lifespan regulation in vivo because of its short lifespan, genetic tractability, transparency, conserved autophagy machinery, and powerful tools for tissue- and age-specific analyses.

### 2.2. Autophagy as a Conserved Driver of Lifespan Extension in C. elegans

**Figure 2:**
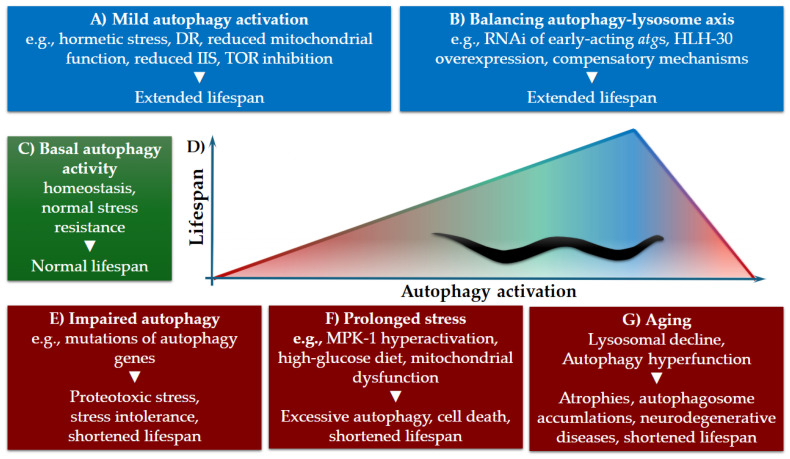
Optimal autophagy activity determines lifespan in *C. elegans*. (**A**) Mild autophagy activation induced by hormetic stressors, such as dietary restriction (DR), enhances proteostasis and stress resistance and extends lifespan. (**B**) Genetic interventions targeting early autophagy components or transcriptional regulators can restore the balance between autophagy induction and lysosomal degradation and partially enhance lifespan. (**C**) Basal autophagy maintains cellular homeostasis and normal longevity. (**D**) In the relationship between autophagy activity and lifespan, lifespan reaches a maximum at mild (or moderate) levels of autophagy activation. (**E**) Impaired autophagy leads to proteotoxic stress and a shortened lifespan. (**F**,**G**) Aging or chronic stress can drive excessive autophagy due to cellular damage or lysosomal dysfunction, resulting in autophagosome accumulation and reduced lifespan. Color coding indicates the impact of autophagy on lifespan in *C. elegans*: green, normal; blue, extended; red, shortened.

*C. elegans* has become an effective model for investigating the genetic and molecular mechanisms that regulate lifespan. The first long-lived mutant identified in this organism carried the *daf-2* (e1370) allele, which encodes a partial loss-of-function variant of the insulin/IGF-1 receptor [26]. Meléndez and colleagues demonstrated that the autophagy gene *bec-1* (homologous to BECN1/ATG6) is essential for the extended lifespan observed in *daf-2* mutants (Figure 2A) [55]. This finding was later supported by studies showing that the inactivation of *atg-18*, *unc-51*, and *bec-1*, or the RNAi-mediated silencing of *atg-7* and *atg-12*, partially suppresses the longevity phenotype of *daf-2* mutants (Table 1) [16,19].

Autophagy is required for lifespan extension across multiple genetically and environmentally induced longevity pathways in *C. elegans*. Beyond reduced insulin/IGF-1 signaling, dietary restriction, TOR inhibition, and mild mitochondrial dysfunction all extend lifespan in an autophagy-dependent manner (Figure 2A) [16]. For example, the lifespan extension observed in *let-363*/TOR mutants, *eat-2* dietary-restriction models, and mitochondrial mutants such as *clk-1* or *atp-3* are suppressed by genetic or RNAi-mediated inhibition of core autophagy genes, including *bec-1*, *unc-51*, and *atg-18* [16,22,23,56,57]. These studies indicate that increased autophagic activity is functionally required for lifespan extension (Table 1).

Many of the cytoprotective mechanisms of autophagy are concentrated in the intestine. Intestine-specific overexpression of the Kruppel-like transcription factor *klf-3*, which regulates cell proliferation and metabolic homeostasis, promotes autophagy by upregulating autophagy-related genes and thereby extends lifespan [58]. In addition, the paracaspase MALT-1, known for its proinflammatory role in mammals, was recently identified as a negative regulator of the early steps of autophagy in *C. elegans*. Loss of *malt-1* enhances lifespan via enhanced activation of the autophagy in the intestine, an effect reversed by RNAi-mediated knockdown of *atg-13*, *bec-1*, and *lgg-2* (Table 1) [59].

Notably, elevated autophagic activity in the intestine leads to the formation of tubular lysosomes (TLs), a specialized lysosomal network, necessary for the lifespan extension of *eat-2* mutants [60]. Although TL formation alone is not sufficient to extend lifespan, their abundance strongly correlates with improved healthspan. This suggests that TLs play a role in maintaining cellular homeostasis during aging (Figure 2B) [60,61]. Moreover, pexophagy—the selective degradation of peroxisomes—becomes more active in the intestine of young adults and starved worms. In *prx-11* mutants, where this process is impaired, lifespan is markedly shortened, underscoring the physiological relevance of peroxisome turnover [62]. These findings emphasize that the role of autophagy is highly tissue-dependent and must be studied with appropriate tissue-specific resolution.

Beyond endogenous regulatory circuits, autophagy can be directly modulated through targeted genetic manipulation. Overexpression of the transcription factor HLH-30/TFEB and the selective autophagy receptor SQST-1/p62 has been shown to extend the lifespan of *C. elegans* by activating autophagy and improving protein homeostasis (Table 1) [63,64,65]. In addition, external environmental factors—such as low-temperature conditions (under 15 °C), dietary exposure to specific amino acids, and in response to stress modulated by *microRNA-34*—can dynamically modulate autophagy and thereby influence the lifespan of *C. elegans* [66,67,68].

Mild, transient heat stress during early adulthood activates the heat-shock response via HSF-1. This stress also stimulates HLH-30/TFEB-dependent autophagy, restoring proteostasis and enhancing lifespan (Figure 2B) [69]. This hormetic effect exemplifies how temporal stress can beneficially activate autophagic activity and counteract age-related proteotoxic damage, which illustrates that maintaining optimal autophagic flux is essential for sustaining proteostasis and delaying the aging process [13,70].

**Table 1:** Beneficial and context-dependent effects of autophagy on lifespan regulation in *Caenorhabditis elegans.* The table summarizes autophagy-modulating conditions, regulators, tissue specificity, and aging markers used.

Condition	Activation Trigger	Autophagy Gene Studied	Gene Manipulation	Tissue	Aging Marker	Ref.
Beneficial effects of autophagy						
TOR pathway inhibition	Genetic triggers	*bec-1*, *unc-51*, *atg-18*	Mutants	Multiple tissues	No	[16]
Reduced insulin/IGF-1	Genetic triggers	*bec-1*	RNAi ^1^	Multiple tissues	No	[55]
Mitochondrial dysfunction	Impaired respiration	*unc-51*, *bec-1*, *atg-18*	Mutants	Multiple tissues	No	[16]
Dietary restriction	Mild stress activation	*atg-18*	RNAi ^1^	Intestine	Intestinal barrier function	[48]
MALT-1 inhibition	Direct autophagy activation	*lgg-2*, *atg-13*, *bec-1*	RNAi	Intestine	Pharyngeal pumping	[59]
SQST-1/p62 overexpression	Autophagy activation	*sqst-1*	Overexpression	Neurons	Proteostasis	[63]
Neuronal lysosomal capacity	Autophagy-flux activation	*hlh-30*	Overexpression	PVD dendrite	Dendrite integrity	[65]
Normal homeostasis	Basal	*bec-1*, *unc-51*, *atg-18*	Mutants	Multiple tissues	Lipofuscin, locomotion	[16]
Prolonged-stress-induced overexpression of autophagy						
Proteotoxicity in *mec-4*(*d*) mutants	Proteotoxic stress	*bec-1*, *unc-51*	Mutants	Neurons	Cell death assay	[71]
Constitutive MPK-1 activation	Starvation	*bec-1*	RNAi ^1^	Pharynx	Pharyngeal muscle integrity	[72]
*sgk-1* inhibition	mPTP activation	*bec-1*	RNAi ^1^	Multiple tissues	Mitochondria permeability	[73]
High-glucose diet	Metabolic stress	*hlh-30*	Mutants	Intestine	No	[74]
Age-dependent negative effects of autophagy						
Neuronal dysregulation	Proteotoxic stress	*bec-1*, *vps-34*	Tissue-specific RNAi	Neurons	Pharynx, muscle, neuron integrity	[18]
Intestinal hyperfunction	Excessive autophagy	*atg-13*	RNAi ^1^	Intestine	Various pathologies	[17]
Lysosomal damage	Excessive autophagy	Multiple *atg*s	Mutants	Multiple tissues	No	[75]

^1^ RNAi in *C. elegans* is typically inefficient in neurons because of limited dsRNA transport and processing, which may affect conclusions regarding neuronal autophagy functions [48].

### 2.3. Detrimental Effects of Excessive Autophagy in C. elegans

While autophagy overactivation is crucial for cytoprotection under hormetic stress, several studies indicate that its persistent hyperactivation can overwhelm cellular degradative capacities [76]. This excessive autophagy leads to deleterious effects, including cellular damage or death [3,77]. Toxic mutations affecting MEC-4, DEG-1, and DEG-3 ion channels induce neurodegeneration through enhanced cellular stress and excessive autophagy [71,78]. Genetic inhibition of key autophagy genes, such as *unc-51*, *bec-1*, and *lgg-1*, suppresses neuronal death, demonstrating that autophagy is not always protective—instead, it can actively contribute to neuronal degeneration [71].

The mitogen-activated protein kinase MPK-1/MAPK functions in the pharyngeal muscles of *C. elegans* during starvation [79]. In *mpk-1* mutants, both autophagy levels and lifespan are diminished during regeneration after refeeding. While MPK-1′s direct role in modulating autophagy remains undemonstrated, constitutive MPK-1 activation is associated with excessive autophagy and pharyngeal tissue damage, resulting in lethality [72].

Moreover, prolonged opening of the mitochondrial permeability transition pore (mPTP) also leads to mitochondrial dysfunction and cell death (Figure 2F) [73]. Zhou et al. (2019) showed that increased autophagy contributes to a shortened lifespan in a *C. elegans* strain lacking SGK-1, the main metabolic regulator downstream of TOR signaling [73,80]. This effect occurs in the context of impaired mitochondrial homeostasis and increased mPTP opening. Suppression of autophagy via RNAi (e.g., *bec-1*, *lgg-1*, and *unc-51*) can restore or partially rescue this phenomenon [73].

Furthermore, under a high-glucose diet, HLH-30-dependent hyperactivation of autophagy leads to lipid dysregulation and a reduced lifespan. Inhibition of HLH-30 suppresses autophagy and restores longevity (Table 1, Figure 2B) [74]. In conclusion, chronic autophagy in diverse tissues and stress contexts can lead to the accumulation of cellular damage or even necrotic cell death.

### 2.4. Antagonistic Pleiotropy and Age-Dependent Reversal of Autophagy Function

Several lines of evidence suggest that autophagy genes can negatively affect the lifespan of *C. elegans*. In contrast to the shortened lifespan observed in *atg-18* mutant animals, loss of *epg-6* (a paralog of *atg-18*) extends lifespan; this is despite both genes contributing to the early steps of autophagosome formation [81]. EPG-6 functions as a PI3P effector that regulates the progression from omegasomes to autophagosomes [82,83]. ATG-18 plays broader, pleiotropic roles, including metabolic regulation and dauer fat accumulation [81]. These observations indicate that distinct autophagy genes can have non-equivalent functions in autophagosome formation and metabolism.

Several subsequent studies demonstrated that even the same autophagy genes can exert opposing effects on lifespan depending on the timing of their inactivation. The first direct evidence demonstrating that autophagy genes with an overall protective role can become detrimental later in life was provided by Hashimoto et al. (2009) [84]. Although lifelong autophagy inactivation reduces lifespan, RNAi silencing of certain autophagy genes, such as *unc-51*, *bec-1*, *atg-7*, and *atg-9*, in young adults slightly enhances it (Figure 2B) [84]. This effect is independent of longevity pathways, such as dietary restriction, insulin/IGF-1 signaling, and reduced mitochondrial respiration. However, the TOR signaling pathway contributes partially to the extension of lifespan, caused by the silencing of autophagy genes in adults [84].

These findings raise an important mechanistic question. How can inhibition of the same process reduce lifespan in one context, yet extend it in another? One possible mechanism involves age-related hyperfunction of intestinal autophagy. In *C. elegans*, yolk proteins are produced via autophagy conversion of intestinal tissue, partly regulated by the transcription factor PHA-4 [85]. In post-reproductive *C. elegans*, continued gut-to-yolk biomass conversion redirects intestinal resources toward yolk production. This leads to intestinal atrophy and ectopic yolk-lipid accumulation that contributes to systemic aging pathologies. Mechanistically, this reflects a failure to downregulate reproduction-associated autophagy after fulfilling its physiological role. Excessive vitellogenesis drives progressive intestinal atrophy and accumulation of pseudocoelomic lipoprotein pools (PLPs), a form of senescent obesity (Figure 2G) [17]. Post-reproductive inhibition of autophagy by RNA interference targeting early-acting autophagy genes (e.g., *atg-13*) suppresses yolk overproduction, alleviates intestinal degeneration, and significantly extends lifespan (Table 1) [17]. These findings demonstrate that late-life intestinal autophagy is a detrimental process rather than a protective recycling mechanism. This supports the theory of antagonistic pleiotropy, which states that mechanisms that enhance early-life reproductive fitness can also drive late-life degeneration [18].

The antagonistic effects of autophagy are not restricted to the intestine. Neuron-specific RNAi silencing of core autophagy genes, such as *bec-1* and *epg-8*, in adults extends lifespan by up to ~30% in *C. elegans* (Table 1) [18]. To distinguish between systemic and neuron-specific effects of autophagy, RNAi experiments were performed using both an *rrf-3* mutant background and a *sid-1* mutant strain with neuron-specific SID-1 expression. In addition, inhibition of autophagy in neurons improved motor function and preserved muscle integrity in a non-cell-autonomous manner (Figure 2G) [18].

According to these examples, there are two distinct mechanisms by which sustained autophagic flux can be detrimental to aged animals. First, in the post-reproductive intestine, autophagy contributes directly to tissue atrophy by degrading limited cytoplasmic components to support yolk production [17]. Intestinal lysosomal capacity can partially adapt to increased autophagosome formation by remodeling the lysosomal network; however, this compensation may ultimately become insufficient or maladaptive (Figure 2B) [61].

Second, although neuronal autophagy functions as a protective mechanism, impaired flux and accumulation of undegraded autophagosomes occur as lysosomal degradative capacity declines with age [75,86]. In both tissues, an imbalance between autophagosome formation and lysosomal clearance results in cellular dysfunction and degeneration (Figure 2) [9].

Post-reproductive inhibition of early-acting autophagy genes reduces autophagosome formation, alleviates flux overload, and significantly mitigates tissue degeneration. These findings suggest that, in late life, sustained neuronal autophagy may represent a maladaptive process whose attenuation promotes longevity and functional health, highlighting the tissue- and age-specific antagonistic roles of autophagy with potential relevance for neurodegenerative disease intervention (Figure 2) [18].

### 2.5. Spatiotemporal and Compensatory Regulation of Autophagy During Aging

Building on the observations described above, autophagy exhibits context-dependent effects on aging. In *C. elegans*, basal autophagy supports development and cellular homeostasis. However, its chronic dysregulation can lead to cellular dysfunction, atrophy, tissue degeneration, and reduced lifespan [17,87]. Lifespan optimization likely depends on the spatiotemporal and targeted control of autophagy. Inhibition of the early steps of autophagy in post-reproductive animals, especially in neurons and the intestine, has been shown to improve overall health and longevity [17,18,84]. However, targeting early-acting autophagy genes that play roles in multiple cellular processes, such as *lgg-1*, may still have harmful effects [17,88].

Among the different tissues, the intestine emerges as a central hub for coordinating systemic aging processes in *C. elegans*. As a multifunctional organ responsible for metabolism, innate immunity, and stress responses, the intestine integrates various longevity pathways [89]. Several long-lived mutants, including those affecting insulin signaling, dietary restriction, or mitochondrial respiration, require intestinal autophagy for lifespan extension [48]. Age-related intestinal pathologies, such as yolk overproduction and lipoprotein accumulation, further highlight the gut as a primary site of aging-related deterioration [17]. This observation suggests that tissue-specific modulation of autophagy, especially in the intestine, may be a promising strategy for enhancing longevity in *C. elegans*.

In certain contexts, autophagy inhibition triggers compensatory mechanisms to preserve proteostasis and organismal health, indicating the presence of compensatory proteostasis mechanisms. For example, neurons under proteotoxic or mitochondrial stress and impaired lysosomal activity increase exopher biogenesis. Exophers extrude aggregates and organelles from the cell, which correlates with better neuronal function [90,91]. Neuronal RNAi (achieved through transgenic expression of *sid-1* in neurons) of autophagy genes acting in early steps of the process, such as *unc-51*, *atg-13*, *atg-7*, *atg-4.1*, and *lgg-1*, leads to enhanced exopher biogenesis in neurons. These exophers facilitate the removal of toxic protein aggregates, such as polyglutamine polypeptides, thereby reducing proteotoxic stress and contributing to improved neuronal function and longevity (Figure 2) [90].

Besides neurons, pharyngeal muscles also exhibit a protective “safety mechanism” that suppresses protein aggregation when core degradation pathways are compromised. This safety mechanism depends on macroautophagy-independent selective lysosomal degradation [92]. Moreover, selective chaperone-mediated autophagy (CMA) has recently been identified in *C. elegans* and shows strong cross-regulation with macroautophagy under starvation conditions in different cell types [93]. However, other possible compensatory mechanisms, including canonical endosomal microautophagy, have not been established in *C. elegans* [93,94]. Compensatory activation of alternative clearance mechanisms may reflect another adaptive response to declining autophagic efficiency with age, mitigating the deleterious effects of persistent or dysfunctional autophagy.

Declining lysosomal capacity, particularly in neuronal cells, has profound consequences for cellular homeostasis and is closely linked to the development of neurodegenerative diseases. In aged or chronically stressed cells, lysosomal function becomes compromised, characterized by impaired acidification, defective transport, and reduced fusion of autophagosomes and endosomes with lysosomes. Consequently, autophagic flux is disrupted, leading to autophagosome accumulation [95,96].

Recent work by Murley et al. 2025 demonstrated that macroautophagy can itself induce lysosomal damage, even in young, arrested larvae, which impedes quiescent cell re-entry [75]. Moreover, Zhong et al. 2025 [65] found that in *C. elegans* neurons, HLH-30 actively expands lysosomal degradative capacity in early adulthood, and this capacity declines with age. Loss of HLH-30 leads to impaired proteostasis and accelerated dendritic degeneration, highlighting lysosomal dysfunction as a key driver of age-associated neuronal decline (Figure 2B) [65]. These results support the notion that maintaining lysosomal competence, rather than indiscriminately enhancing autophagy, can be critical for maintaining tissue health during aging.

It is worth noting that, in this review, we focused primarily on the effect of modulating autophagy on lifespan. Although lifespan correlates with healthspan, it does not always accurately capture the functional and physiological aspects of aging. In *C. elegans*, several well-established approaches enable direct assessment of healthspan, including the quantification of lipofuscin accumulation as a marker of cellular aging, measurements of locomotion and paralysis, pharyngeal pumping assays, and behavioral or neuromuscular tests that monitor tissue functionality [97,98,99]. Incorporating such parameters can refine our understanding of how autophagy influences not only lifespan but also the quality of aging in this model (Table 1).

## 3. Relevance to Human Disease

The metaphor of the “double-edged sword” reflects its paradoxical function in human diseases, particularly in cancer. Autophagy can act both as a tumor suppressor and as a survival mechanism for malignant cells [100,101]. For instance, in KRAS-mutant lung cancer, autophagy supports tumor cell survival under metabolic and therapeutic stress, whereas inhibition of ULK1 sensitizes these cells to stress [102].

Additional support that the hyperfunction of autophagy turns to malfunction comes from metabolic and toxicological studies. In β-cells where ferroptosis—a type of regulated cell death—can be induced by arsenite (NaAsO_2_), the mitochondria–ROS–autophagy–ferritin axis was found to drive the process through excessive autophagy. Inhibition of ferritin degradation by chloroquine showed a cytoprotective effect [103]. Similarly, empagliflozin attenuates excessive autophagy via AMPK/GSK3β signaling in high-glucose-treated cardiomyocytes and improves cardiac dysfunction in diabetic mice, indicating that induction of autophagy can be detrimental [104].

Moreover, in neurodegenerative disorders such as Alzheimer’s and Parkinson’s diseases, pathology often arises from the defects in autophagy flux and the accumulation of cytotoxic aggregates [105]. Accordingly, therapeutic strategies increasingly target defined blockage points, for example, by restoring lysosomal function to re-establish autophagic flux [96,106,107,108,109].

In contrast, a second therapeutic direction, aligned with findings from *C. elegans*, involves the directed inhibition of early autophagy steps. Alzheimer’s disease was linked to hyperactive secretory autophagy, showing that its excessive activation in the brain promotes neuroinflammation, hippocampal atrophy, and neurodegeneration [110]. According to these observations, in mammalian cells, excessive or misregulated autophagy can drive pathology, and the modulation of early steps or autophagosome–lysosome fusion can be beneficial.

However, in most inflammatory diseases, neurodegenerative diseases, or intracellular pathogen infections, genetic inhibition of core autophagy genes, such as ATG5, BECN1, or ATG16L1, reveals predominantly protective roles of autophagy [111]. Based on these facts, complete or chronic inhibition of autophagy is generally detrimental, particularly in post-mitotic tissues [18,111].

Hormetic interventions, such as intermittent fasting or mild heat-stress response, have been shown to modestly enhance autophagy and improve proteostasis in mammals, echoing the beneficial effects of moderate autophagic activation observed in *C. elegans* [76,112]. Overall, human and *C. elegans* studies confirm that both insufficient and excessive autophagy can disrupt cellular homeostasis and contribute to disease, highlighting the importance of maintaining a balanced autophagic activity for healthy aging [113]. Notably, several human orthologs of key regulatory factors remain poorly characterized. Comparative studies across species may help bridge current gaps in our understanding of autophagy and aging.

## 4. Conclusions

Recent studies in *C. elegans*, summarized in this review, support the view of autophagy as a context-dependent, “double-edged” regulator of aging rather than a uniformly protective process. Its effects are determined by timing, tissue specificity, physiological state, and cellular stress levels, underscoring the need for more precise modulation [51,113]. Advances in genetic tools, such as AID-based protein depletion, enable reversible, tissue-specific, and temporally controlled manipulation of autophagy, even in post-reproductive stages, overcoming limitations of RNAi approaches—particularly in neurons [50,114]. Integrating these strategies with systematic healthspan measurements will be essential to understand the functional consequences of autophagy modulation, which may be reflected not only in lifespan but also in tissue integrity and neuronal functions such as learning and memory [53,99,115]. Ultimately, these approaches should clarify how compensatory responses interact with autophagy during aging, revealing when adaptive mechanisms are more beneficial than sustained autophagy activity and when autophagy itself becomes detrimental [52].

## Data Availability

No new data were created or analyzed in this study. Data sharing is not applicable to this article.

## Abbreviations

The following abbreviations are used in this manuscript:

AID	Auxin-inducible degradation
AD	Alzheimer’s disease
*atg*s	Autophagy-related genes
*C. elegans*	*Caenorhabditis elegans*
DR	Dietary restriction
ER	Endoplasmic reticulum
FOXA	Forkhead box A
HLH-30	Helix–loop–helix protein 30
IGF-1	Insulin growth factor 1
mTORC1	Mammalian target of rapamycin complex 1
PHA-4	Pharynx defective protein 4
PLPs	Pseudocoelomic lipoprotein pools
RNAi	RNA interference
ROS	Reactive oxygen species
SID	Systemic RNA interference-defective
TFEB	Transcription factor EB
TOR	Target of rapamycin
TLs	Tubular lysosomes
WIPI	WD-repeat protein Interacting with Phosphoinositides
